# Metastasis from tongue squamous cell carcinoma to the kidney

**DOI:** 10.4322/acr.2021.257

**Published:** 2021-03-12

**Authors:** Hanan S. Elsarraj, Sidrah Khawar, Ameer Hamza

**Affiliations:** 1 University of Kansas Medical Center, Department of Pathology and Laboratory Medicine, Kansas City, KS, USA; 2 University of Texas, MD Anderson Cancer Center, Department of Pathology, Houston, TX, USA

**Keywords:** Neoplasm Metastasis, Oropharynx, Tongue, Kidney

## Abstract

Metastasis to the kidney from other primary sites is extremely rare. Previous studies reported the lung as the most common primary site. Distant metastasis from the tongue to the kidney is exceedingly rare. Herein, we describe a case of metastatic squamous cell carcinoma to the kidney in a 71-year-old male with a detailed discussion of differentiating it from potential mimickers. The patient underwent a total glossectomy and bilateral cervical lymph node dissection. A diagnosis of well-differentiated squamous cell carcinoma of the tongue was rendered and the tumor was staged pT3 pN3b. Within two years of initial presentation, the patient developed widely metastatic disease, including pulmonary nodules, renal masses, left adrenal mass, and pancreatic mass. Accurate diagnosis of a secondary involvement of the kidney by a metastatic tumor requires the appropriate correlation of clinical and imaging findings as well as morphologic and immunohistochemical clues.

## INTRODUCTION

Squamous cell carcinoma (SCC) is the most common oropharyngeal malignancy. It accounts for over 90% of all oropharyngeal malignancies. Within the oropharynx, 25-40% of these tumors involve the tongue.^1^ 5-year survival rate is 50-55%, owing primarily to metastatic disease to regional and distant sites. The risk of distant metastasis is approximately 30% for patients with lymph node metastasis, and typically occurs 9-12 months after initial diagnosis. The most common sites of distant metastatic involvement are the lungs and bones.^1^^,^^2^ Metastasis of squamous cell carcinoma of the tongue to kidney is exceedingly rare, with only two reported cases in the literature.^3^^,^^4^

Herein, we describe a case of metastatic squamous cell carcinoma to the kidney in a 71-year-old male.

Differential diagnosis with urothelial carcinoma with squamous differentiation and collecting duct carcinoma is also discussed. A review of literature is also provided in the discussion with an emphasis on metastatic tumors to the kidney and metastatic squamous cell carcinoma to the kidney in particular.

## METHOD

We searched PubMed and Scopus databases from their inception (1996 and 2004, respectively) to December 2020, using the keywords “metastasis to kidney”; “secondary tumors of kidney”; “secondary renal neoplasms”; and “metastatic squamous cell carcinoma to the kidney”. This was followed by a manual search of the included references. A total of 18 articles were initially identified. Duplicates were removed, and all articles with primary renal neoplasms were excluded. Five articles are mainly discussed in the review of the literature.

## CASE REPORT

**Figure 1:**
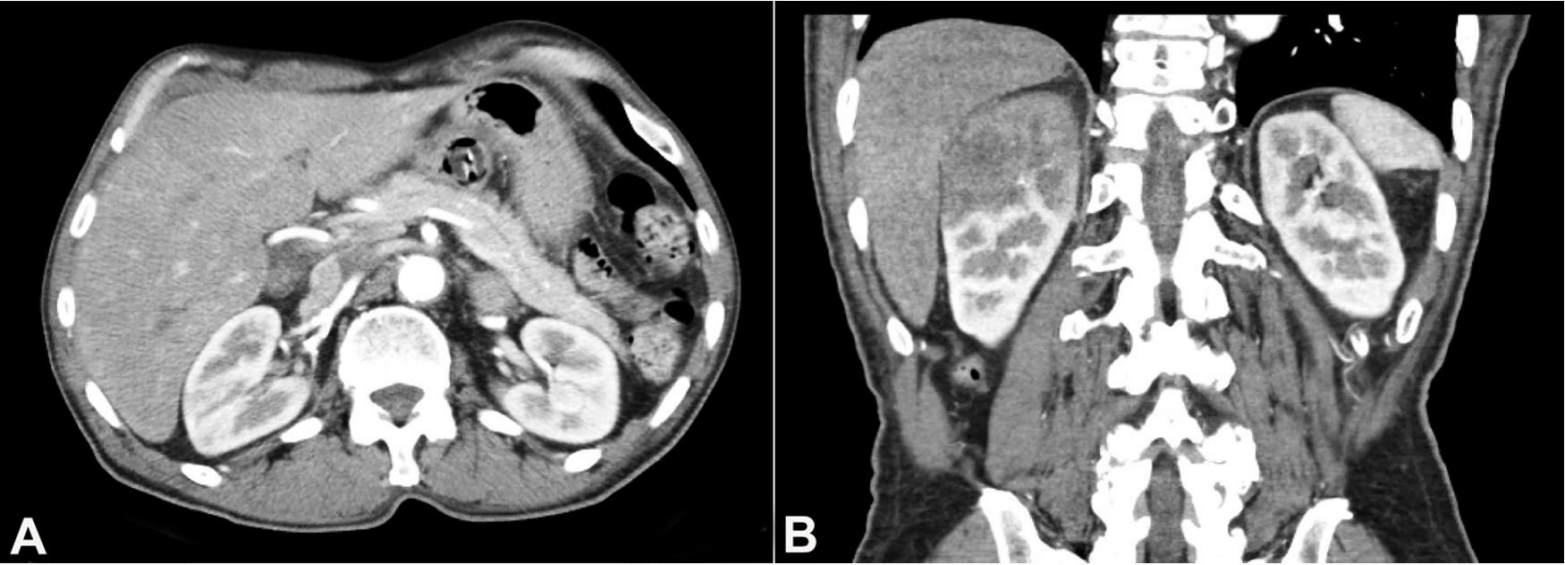
CT scan demonstrating ill-defined low-attenuation masses within the superior pole and mid-region of the right kidney.

A 71-year-old male initially presented with the chief complaint of oral cavity mass. Notably, he had 30 pack-years smoking history. CT neck demonstrated right-sided tongue and floor of mouth mass, and possible metastatic disease within two right-sided cervical lymph nodes. He underwent a PET scan, which confirmed a hypermetabolic mass in the oral cavity and ipsilateral nodal disease. No sign of distant disease was identified at that time. He underwent a total glossectomy and bilateral cervical lymph node dissection. Pathology revealed an invasive, keratinizing, well-differentiated squamous cell carcinoma, with and metastatic carcinoma involving bilateral level 2-4 lymph nodes. A p16 immunohistochemical stain was negative. The tumor was staged as pT3 pN3b. Subsequently, the patient received adjuvant therapy. He presented for his regular follow-up 18 months after completion of adjuvant chemotherapy and radiation. Surveillance chest computed tomography (CT) scan revealed a right upper lobe endobronchial lesion and right hilar lymphadenopathy. The biopsy showed metastatic SCC in the right upper lobe of the lung. Imaging performed five months later showed newly developed pulmonary nodules representing additional sites of metastatic disease. In addition, abdominal and pelvic CT demonstrated newly developed renal masses, left adrenal mass, and pancreatic mass, also likely representing metastatic disease (Figure 1).

Repeat PET imaging showed interval increase in size and number of multiple bilateral pulmonary nodules, which demonstrated hypermetabolic activity. The left upper lobe nodule measured 1.1 cm, previously 0.6 cm, with max SUV 4.0. Hypermetabolic bilateral hilar, mediastinal, and retrocrural lymphadenopathy was also noted. Furthermore, the abdomen/pelvis showed hypermetabolic infiltrating masses within the superior pole of the right kidney, demonstrating max SUV 31.5 and a hypermetabolic left adrenal mass, consistent with metastatic disease. In order to confirm the radiologic impression, a biopsy of the renal mass was performed.

**Figure 2:**
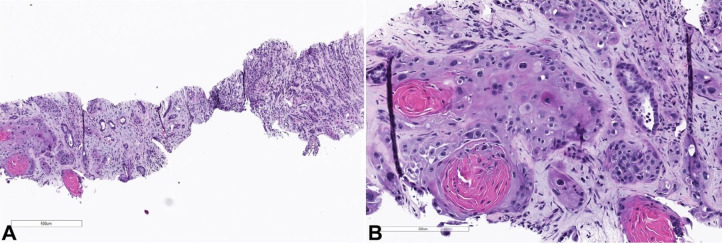
Low magnification image showing the tumor infiltrating the renal parenchyma (H&E, 40x); high magnification showing cytologic details of the tumor cells (H&E 200x).

Microscopic examination of the CT-guided biopsy of the renal mass revealed infiltrative growth of malignant cells with abundant eosinophilic cytoplasm, areas of keratinization, and large, vesicular nuclei (Figure 2).

**Figure 3:**
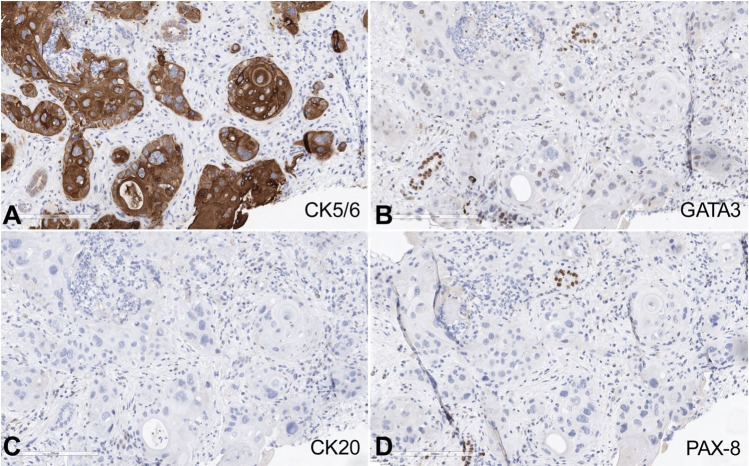
Immunohistochemical staining for anti-CK5/6 (A), anti-GATA3 (B), anti-CK20 (C), anti-PAX8 (D).

Immunohistochemical studies showed the tumor cells to be diffusely and strongly positive for CK5/6 and negative for PAX8 and CK20 (Figure 3). GATA3 was predominantly negative with focal weak positivity in some cells. The morphology and immunohistochemical profile were interpreted to be consistent with metastatic squamous cell carcinoma involving the renal parenchyma.

The patient subsequently completed three cycles of chemotherapy and immunotherapy. Three months have elapsed since renal involvement was noted on imaging studies, and the patient is alive with widely metastatic disease. He is scheduled for a follow-up CT for assessment of metastatic disease and treatment response.

## DISCUSSION

Although metastases from a primary renal tumor are fairly common, metastasis to the kidney from other primary sites is extremely rare. A comprehensive analysis of 151 cases of secondary renal tumors revealed that the most common primary sites include the lung, gastrointestinal tract, breast, and thyroid.^5^ The median patient age was found to be 56.7 years, and metastases to the kidney were solitary in 77.5% of cases.^5^ Another series of 43 surgical pathology cases found similar results with the median age of 61 years and male to female ratio of 1:1.^6^ The most common primary sites in this series included lung, breast, and female genital tract.^6^ Both studies mentioned the head and neck region/ear, nose, and throat (ENT) as one of the primary sites; however, the specific site within the head and neck region or specific tumor was not mentioned. Our patient was 71-years-old and had two foci of metastatic disease in his right kidney. The mainstay of diagnosis is imaging studies. Based on imaging findings, renal masses can be categorized as benign, primary renal tumor, metastasis, and uncertain. In the study by Zhou, C. et al.,^5^ the radiology-pathology concordance was 51%, whereas in the study by Wu AJ et al.^6^ the concordance was 54%.^5^^,^^6^ In our patient, the radiologic impression was that of metastatic disease. Secondary involvement of the kidney usually occurs in the setting of widely metastatic disease.^5^^,^^7^ Solitary involvement of the kidney is less common; however, no other site of metastatic disease was identified at the time of renal involvement in 37% of patients in the study by Wu AJ et al.^6^ Solitary involvement of the kidney represents an opportunity for therapy with curative intent. In the study by Zhou C et al.^5^ surgery with curative intent was performed in 21 patients.^5^ Our patient was not a suitable candidate for curative surgery owing to widely metastatic disease.

In the study by Wu AJ et al.,^6^ eight cases (19%) were squamous cell carcinomas; however, for all eight cases, the primary site was lung.^6^ In cases of a secondary involvement of the kidney by a squamous cell carcinoma, it is prudent to rule out other potential mimickers such as a urothelial carcinoma with squamous differentiation and collecting duct carcinoma. The presence of a conventional urothelial carcinoma or an in-situ component favors the diagnosis of urothelial carcinoma; however, in the absence of these features and in the setting of extensive squamous differentiation, the distinction is extremely challenging in a core biopsy as well as a resection specimen. Immunohistochemistry is potentially useful but not entirely diagnostic because of overlapping features. Squamous cell carcinomas are usually CK20 negative, whereas a subset of urothelial carcinomas with squamous differentiation are positive. CK7 usually shows positivity in urothelial tumors and is predominantly negative in squamous cell carcinoma. Strong and diffuse GATA3 positivity favors a urothelial primary; however, a weak and patchy GATA3 expression has been reported in squamous cell carcinomas.^8^^-^^10^ The distinction from collecting duct carcinoma can be challenging on morphologic grounds alone. Immunohistochemical stains, including PAX-8 and CK5/6, can be helpful in this distinction. Our case was strongly positive for CK5/6 and negative for PAX8, and CK20. GATA3 was negative with focal weak, non-specific positivity in our case. Accurate diagnosis requires the correlation of all the relevant information, including the clinical history, radiologic findings, and morphologic and immunohistochemical features. Known history of a primary tumor is an extremely helpful clue. In the study by Wu AJ et al.,^6^ 88% of patients had a known history of primary disease, and in an additional 9%, the primary tumor and metastasis were diagnosed concurrently.^6^ The time interval between the initial diagnosis and the secondary involvement of the kidney is variable. Wu AJ et al.^6^ reported the median time interval to be 3.1 years (range 0 to 21.6 years).^6^ The time interval was greater than 10 years in 19% of their patients.^6^ In the case reported by Singh GK et al.,^3^ the time interval was 18 months, while in the case reported by Thyavihally Y et al.,^4^ the time interval was 4 months.^3^^,^^4^ Our patient developed lung metastasis 18 months after completion of therapy for the primary tumor and 5 months later developed widely metastatic disease, including the kidney involvement.

In patients with oropharyngeal squamous cell carcinoma, the risk of distant metastasis is approximately 30% for patients with lymph node metastasis.^2^ The 5-year relative survival for distant metastatic oral cavity and pharynx cancer is 40.1%.^11^ In the study by Zhou C et al.,^5^ the median overall survival (OS) from the time of renal metastasis diagnosis was 1.13 years.^5^ The OS from the time of renal metastasis diagnosis was 2.24 years for patients who underwent renal surgery compared to 0.72 years or patients who did not undergo renal surgery (log-rank P < 0.001).^5^ Our patient was alive with the widely metastatic disease three months after renal involvement was noted on imaging studies.

## CONCLUSION

Secondary involvement of the kidney by primary tumors of different organs is rare. Appropriate clinicopathologic correlation is immensely helpful in making the correct diagnosis. Median OS from the time of renal metastasis is just over a year, and surgical intervention may be helpful in improving the OS to over 2 years.

## References

[1] Gorsky M, Epstein JB, Oakley C, Le ND, Hay J, Stevenson-Moore P (2004). Carcinoma of the tongue: a case series analysis of clinical presentation, risk factors, staging, and outcome. Oral Surg Oral Med Oral Pathol Oral Radiol Endod.

[2] Calhoun KH, Fulmer P, Weiss R, Hokanson JA (1994). Distant metastases from head and neck squamous cell carcinomas. Laryngoscope.

[3] Singh GK, Singh P, Yadav V, Shanmuga PB, Periasamy K (2017). Unusual sites of metastases in carcinoma tongue - A case report. OGH reports.

[4] Thyavihally YB, Tongaonkar HB, D’Cruz AK, Chinoy RF (2005). Carcinoma of tongue with solitary metastasis to kidney - case report. Indian J Urol.

[5] Zhou C, Urbauer DL, Fellman BM (2016). Metastases to the kidney: A comprehensive analysis of 151 patients from a tertiary referral centre. BJU Int.

[6] Wu AJ, Mehra R, Hafez K, Wolf JS, Kunju LP (2015). Metastases to the kidney: A clinicopathological study of 43 cases with an emphasis on deceptive features. Histopathology.

[7] Aleong CBA, Baithun S (2000). Secondary neoplasms of the kidney: A clinico-pathological review of 443 cases. J Pathol.

[8] Liang Y, Heitzman J, Kamat AM, Dinney CP, Czerniak B, Guo CC (2014). Differential expression of GATA-3 in urothelial carcinoma variants. Hum Pathol.

[9] Gulmann C, Paner GP, Parakh RS (2013). Immunohistochemical profile to distinguish urothelial from squamous differentiation in carcinomas of urothelial tract. Hum Pathol.

[10] Miettinen M, McCue PA, Sarlomo-Rikala M (2014). GATA3: A multispecific but potentially useful marker in surgical pathology: a systematic analysis of 2500 epithelial and nonepithelial tumors. Am J Surg Pathol.

[11] Surveillance Research Program NCI SEER*Explorer: An interactive website for SEER cancer statistics.

